# An Updated Meta-Analysis of the Associations Between MicroRNA Polymorphisms and Susceptibility to Rheumatoid Arthritis

**DOI:** 10.3389/fphys.2018.01604

**Published:** 2018-11-15

**Authors:** Mi Zhou, Bo Jiang, Mao Xiong, Xin Zhu

**Affiliations:** ^1^Department of Respiratory and Critical Care Medicine, The First Affiliated Hospital of Chongqing Medical University, Chongqing, China; ^2^Department of Urology, The First Affiliated Hospital of Chongqing Medical University, Chongqing, China

**Keywords:** microRNA, single nucleotide polymorphism, rheumatoid arthritis, meta-analysis, systematic review

## Abstract

**Aims:** Rheumatoid arthritis (RA) is characterized by cartilage and bone damage leading to disability. Here, the association between microRNA (miRNA) polymorphisms and susceptibility to RA was evaluated by performing an updated meta-analysis and systematic review.

**Main methods:** An electronic search of databases including PubMed and Embase was performed from inception to December 8, 2017 to retrieve studies investigating the association between miRNA polymorphisms and RA risk. Two reviewers independently screened literature according to the inclusion and exclusion criteria and extracted data. The meta-analysis was conducted using Stata 14.0 software.

**Key findings:** Thirteen case-control studies with 2660 cases and 4098 controls were screened out after a systematic search. One study from the miR-146a rs2910164 G > C polymorphism group and two from the miR-499 rs3746444 T > C polymorphism group were excluded because of deviations from Hardy-Weinberg equilibrium. Pooled analysis demonstrated that miR-146a rs2910164 G > C polymorphism was not significantly associated with susceptibility to RA. However, a significant association was observed between miR-499 rs3746444 T > C polymorphism and RA risk (C vs. T: OR = 1.22, 95% CI = 1.05–1.42, *P* = 0.008; TC vs. TT: OR = 1.26, 95% CI = 1.05–1.50, *P* = 0.011; TC/CC vs. TT: OR = 1.26, 95% CI = 1.07–1.5, *P* = 0.007). Subgroup analysis based on ethnicity showed no significant association between miR-499 T > C polymorphism and susceptibility to RA in the Asian population (*P* > 0.05). However, in Caucasian population, the C allele in the miR-499 T > C polymorphism was a contributor to RA susceptibility in some genetic models (C vs. T: OR = 1.64, 95% CI = 1.28–2.11, *P* < 0.001; TC vs. TT: OR = 1.95, 95% CI = 1.40–2.71, *P* < 0.001; TC/CC vs. TT: OR = 1.96, 95% CI = 1.43–2.69, *P* < 0.001).

**Significance:** The miR-146a rs2910164 G > C polymorphism was not associated with susceptibility to RA. In the Caucasian population, the C allele in the miR-499 T > C polymorphism contributed to RA susceptibility.

## Introduction

Rheumatoid arthritis (RA), one of the most prevalent chronic inflammatory diseases, is characterized by damage to cartilage and bone leading to disability (Smolen et al., 2016). It is a substantial burden for individuals and society, with up to 0.5% of the global population affected (Neogi et al., 2010), and the identification of novel therapeutic strategies to control inflammation and reduce severe consequences is increasingly imperative. The pathogenesis of RA is closely associated with genetic and environmental factors, with the major histocompatibility complex (MHC) region at 6p21 contributing to 30–50% of genetic liability to RA (Yamamoto et al., 2015; Okada et al., 2016; Terao et al., 2016). Cigarette smoking also plays an important role in RA risk (Sokolove et al., 2016).

Rheumatoid arthritis risk was recently shown to be associated with epigenetic regulation including DNA methylation, histone modification, and transcriptional/post-transcriptional regulation (Klein and Gay, 2015). MicroRNAs (miRNAs), which are the most widely studied class of non-coding RNAs, function in the post-transcriptional regulation of gene expression by controlling the translation of mRNA into proteins (He and Hannon, 2004). Moreover, miRNAs play a significant role in regulating biological processes, including proliferation, apoptosis, and development (Esteller, 2011).

A growing body of evidence indicates that single nucleotide polymorphisms (SNPs) in miRNAs may affect RA susceptibility because certain SNPs located in miRNA encoding genes can alter miRNA expression, pri-miRNA and pre-miRNA processing, or miRNA–mRNA interactions (Furer et al., 2010; Dai and Ahmed, 2011).

Ciccacci et al. (2016) reported that the C allele of rs2910164 in miR-146a has a protective role in the pathogenesis of RA. However, Piatek et al. (2016) reported no significant association between miR-146a polymorphisms and RA susceptibility or progression. With regard to miR-499, the effect of C allele of rs3746444 on RA risk is also controversial (El-Shal et al., 2013; Zhang et al., 2013). The association between miRNA polymorphisms and susceptibility to RA remain unclear. Because of increasing number of case-control studies published, an updated meta-analysis is required to evaluate the relationship between miRNA polymorphisms and RA, including subgroup analyses investigating ethnicity-specific effects.

## Materials and Methods

### Literature Search Strategy

A comprehensive search of the PubMed and Embase databases was performed to retrieve studies investigating the association between miRNA polymorphisms and RA susceptibility published in English up to December 8, 2017. The search terms were as follows: “rheumatoid arthritis” “arthritis” “microRNA” “miRNAs” “polymorphism” and “polymorphisms.” Relevant journals and the references of the included studies were also reviewed.

### Inclusion and Exclusion Criteria

The inclusion criteria of our meta-analysis were as follows: (1) case-control studies; (2) investigation of miRNA polymorphisms and RA susceptibility; (3) detailed numbers of alleles and genotypes between cases and controls; (4) full text published in English; (5) control group consistent with Hardy-Weinberg Equilibrium (HWE); and (6) any specific miRNA with at least two case-control studies to evaluate the association between single nuclear polymorphism and RA susceptibility.

The exclusion criteria were as follows: (1) reviews or case reports without controls; (2) no available data reported; and (3) duplicated reports.

### Data Extraction

Two investigators (Zhou and Jiang) screened the literature and extracted data independently according to the inclusion and exclusion criteria. Discrepancies and differences were resolved by a third investigator (Zhu) until consensus was reached. The following data were retrieved: author list, year of publication, ethnicity, sample size, alleles, genotype of each gene polymorphism, and HWE.

### Data Synthesis and Statistical Analysis

The odds ratios (ORs) with their corresponding 95% confidence intervals (CIs) were used to assess the strength of the association between polymorphisms in miRNAs and RA. For miR-146a rs2910164 G > C, the dominant (GC + CC vs. GG), recessive (CC vs. GC + GG), heterozygous (GC vs. GG), homozygous (CC vs. GG), and allelic (C vs. G) genetic models were used to calculate pooled ORs. These models were also used to evaluate the miR-499 rs3746444 T > C polymorphism, including the dominant (TC + CC vs. TT), recessive (CC vs. TC + TT), heterozygous (TC vs. TT), homozygous (CC vs. TT), and allelic (C vs. T) models.

The Chi square-based *Q*-test was used to assess the between-study heterogeneity and a *P*-value of 0.1 was considered to indicate significant heterogeneity among studies (Lau et al., 1997). The I^2^ metric value was also used to describe the proportion of total variation in study estimates due to heterogeneity, with values of 25, 50, and 75% considered as evidence of low, moderate, and high heterogeneity, respectively (Higgins and Thompson, 2002). The pooled OR estimation of each study was calculated by the fixed-effects model (the Mantel–Haenszel method) when the *P*-value was >0.1 and I^2^ was <50% (Mantel and Haenszel, 1959). Otherwise, the random-effects model (the DerSimonian and Laird method) was used (DerSimonian and Laird, 1986). Subgroup analyses according to ethnicity were also performed to calculate ethnic-specific ORs. The HWE was tested for each study by comparing the observed and expected genotype frequencies of the control group (Chi-square test). Publication bias was evaluated with Egger’s linear regression and Begg’s funnel plots (Hayashino et al., 2005). Statistical analyses were performed using the Stata software (version 14.1; Stata Corp., College Station, TX, United States) with a two-sided *P*-value. *P* < 0.05 was considered significant.

## Results

### Study Characteristics

**FIGURE 1:**
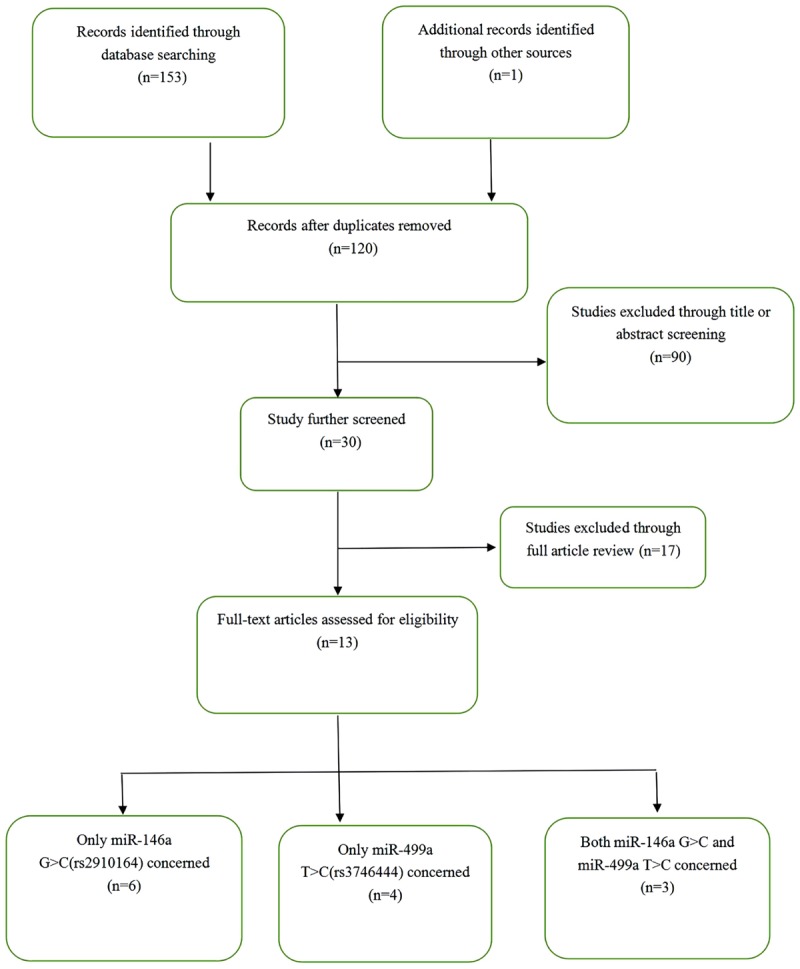
Flow chart showing the detailed steps for literature selection.

The results of the systematic search and selection performed according to the inclusion and exclusion criteria are shown in Figure 1. A total of 13 articles with 2660 cases and 4098 controls were finally included in the meta-analysis (Chatzikyriakidou et al., 2010; Yang et al., 2011, 2016; Jimenez-Morales et al., 2012; El-Shal et al., 2013; Hashemi et al., 2013; Zhang et al., 2013; Zhou X. et al., 2015; Bogunia-Kubik et al., 2016; Ciccacci et al., 2016; Toraih et al., 2016; Fattah et al., 2017; Hassine et al., 2017). The 13 articles comprised 16 studies, including 9 studies on miR-146a rs2910164 and 7 studies on miR-499 rs3746444. Table 1 shows the characteristics of the retrieved studies. Various genotyping methods were used, including PCR-RFLP, TaqMan, and PCR. The genotype distribution of the controls in all studies was consistent with HWE, except one study from the miR-146a polymorphism group (El-Shal et al., 2013) and two from the miR-499 polymorphism group (Hashemi et al., 2013; Toraih et al., 2016).

**Table 1:** The basic characteristics of the retrieved studies.

First author	Year	Country	Ethnicity	Genotyping method	Sample size		Genotypes (case/control)						HWE (P)
					Case	Control	Case			Control			
								
							GG	GC	CC	GG	GC	CC	
**miR-146a G > C**													
Hasssine	2017	Tunisia	Caucasian	PCR-RFLP	165	150	94	63	8	68	69	13	0.441
Ciccacci	2016	Italia	Caucasian	TaqMan PCR	192	298	109	69	14	158	117	23	0.836
Bogunia-Kubik	2016	Poland	Caucasian	PCR–RFLP	111	130	72	32	7	88	36	6	0.361
Zhou	2015	China	Asian	PCR	598	821	114	283	201	151	385	285	0.296
El-Shal	2013	Egypt	Caucasian	PCR-RFLP	217	245	30	103	84	15	119	111	0.021
Hashemi	2013	Iran	Caucasian	T-ARMS-PCR	104	110	57	39	8	64	37	9	0.280
Jimenez-Morales	2012	China	Asian	TaqMan PCR	210	531	102	80	28	236	229	66	0.369
Yang	2011	China	Asian	PCR-RFLP	208	240	28	95	85	30	116	94	0.529
Chatzikyriakidou	2010	Greece	Caucasian	PCR-SSCP	136	147	73	53	10	80	53	14	0.240
							**TT**	**CT**	**CC**	**TT**	**CT**	**CC**	
**miR-499a T > C**													
Fattah	2017	Egypt	Caucasian	PCR-RFLP	100	100	33	53	14	49	41	10	0.742
Yang	2016	China	Asian	TaqMan PCR	386	576	282	99	5	443	125	8	0.807
Toraih	2016	Egypt	Caucasian	TaqMan PCR	95	200	50	15	30	82	68	50	<0.001
El-Shal	2013	Egypt	Caucasian	PCR-RFLP	217	245	113	93	11	167	70	8	0.841
Zhang	2013	China	Asian	MALDI-TOF MS	206	466	159	44	3	346	110	10	0.719
Hashemi	2013	Iran	Caucasian	T-ARMS-PCR	104	110	46	32	26	74	25	11	<0.001
Yang	2011	China	Asian	PCR-RFLP	208	240	159	42	7	182	53	5	0.624


### Meta-Analysis

**FIGURE 2:**
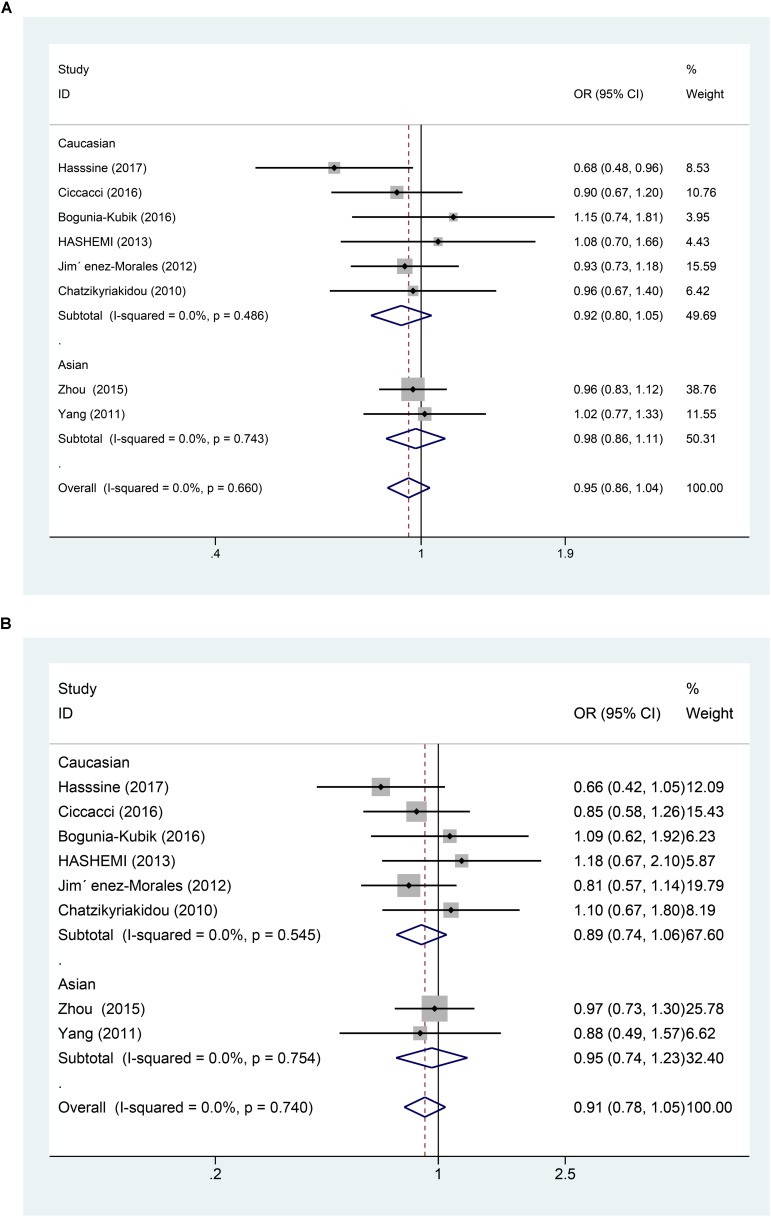
**(A)** Forest plot for association between miR-146a rs302789 polymorphisms and risk of rheumatoid arthritis under allele genetic model (C vs. G) after stratification analysis by ethnicity. **(B)** Forest plot for association between miR–146a rs302789 polymorphisms and risk of rheumatoid arthritis under heterozygote genetic model (GC vs. GG) after stratification analysis by ethnicity.

**FIGURE 3:**
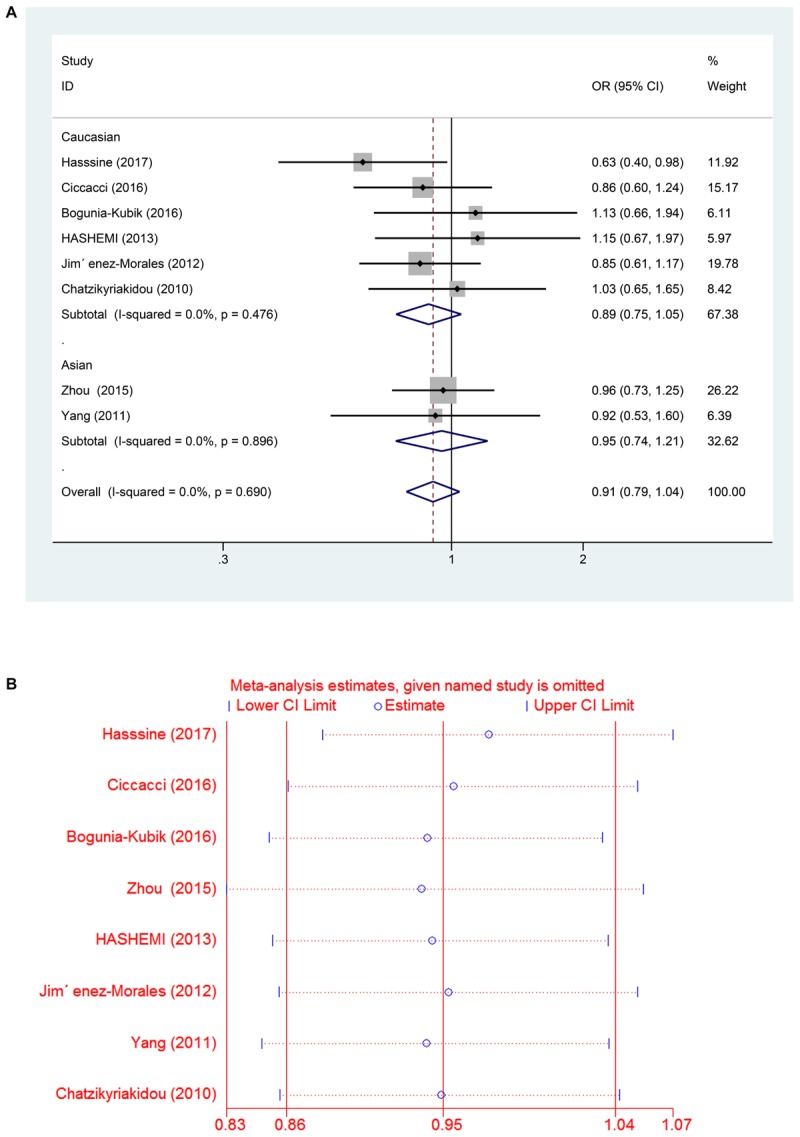
**(A)** Forest plot for association between miR-146a rs302789 polymorphisms and risk of rheumatoid arthritis under dominant genetic model (GC/CC vs. GG) after stratification analysis by ethnicity. **(B)** The sensitivity analysis for studies of the association between miR–146a rs302789 polymorphisms and risk of rheumatoid arthritis under allele genetic model (C vs. G).

In all the populations, the miR-146a rs2910164 G > C polymorphism was not significantly associated with increased susceptibility to RA (C vs. G: OR = 0.95, 95% CI = 0.86–1.04, P_h_ = 0.66; CC vs. GG: OR = 0.91, 95% CI = 0.75–1.12, P_h_ = 0.87; GC vs. GG: OR = 0.91, 95% CI = 0.78–1.05, P_h_ = 0.74; GC/CC vs. GG: OR = 0.91, 95% CI = 0.79–1.04, P_h_ = 0.69; CC vs. GC/GG: OR = 0.97, 95% CI = 0.82–1.14, P_h_ = 0.896). Subgroup analysis based on ethnicity showed no significant association between the miR-146a C/G polymorphism and susceptibility to RA in the Asian and Caucasian populations (*P* > 0.05) (Table 2 and Figures 2A,B, 3A).

**Table 2:** Meta-analysis results of the miR-146a rs3027898 and miR-499a rs3746444 polymorphisms with genetic susceptibility to rheumatoid arthritis.

Variables	*N*	C vs. G			CC vs. GG			GC vs. GG			GC/CC vs. GG			CC vs. GC/GG		
		OR (95% Cl)	P_h_	I^2^(%)	OR (95% Cl)	P_h_	I^2^(%)	OR (95% Cl)	P_h_	I^2^(%)	OR (95% Cl)	P_h_	I^2^(%)	OR (95% Cl)	P_h_	I^2^(%)
**miR-146a G > C**																
Caucasian	6	0.92 (0.80–1.05)	0.486	0.0	0.88 (0.64–1.20)	0.692	0.0	0.89 (0.74–1.06)	0.545	0.0	0.89 (0.75–1.05)	0.476	0.0	0.93 (0.69–1.26)	0.772	0
Asian	2	0.98 (0.86–1.11)	0.743	0.0	0.94 (0.72–1.23)	0.915	0.0	0.95 (0.74–1.23)	0.754	0.0	0.95 (0.75–1.21)	0.896	0.0	0.98 (0.81–1.19)	0.593	0
All ethnicity	8	0.95 (0.86–1.04)	0.660	0.0	0.91 (0.75–1.12)	0.870	0.0	0.91 (0.78–1.05)	0.740	0.0	0.91 (0.79–1.04)	0.690	0.0	0.97 (0.82–1.14)	0.896	0

		**C vs. T**			**CC vs. TT**			**TC vs. TT**			**TC/CC vs. TT**			**CC vs. TC/TT**		
		**OR (95% Cl)**	**P_h_**	**I^2^(%)**	**OR (95% Cl)**	**P_h_**	**I^2^(%)**	**OR (95% Cl)**	**P_h_**	**I^2^(%)**	**OR (95% Cl)**	**P_h_**	**I^2^(%)**	**OR (95% Cl)**	**P_h_**	**I^2^(%)**

**miR-499a T > C**																
Caucasian	2	1.64 (1.28–2.11)	0.740	0.0	2.06 (1.06–3.97)	0.973	0.0	1.95 (1.40–2.71)	0.951	0.0	1.96 (1.43–2.69)	0.977	0.0	1.52 (0.81–2.86)	0.906	0.0
Asian	3	1.04 (0.86–1.25)	0.351	4.4	1.03 (0.53–2.01)	0.598	0.0	1.05 (0.85–1.29)	0.293	18.5	1.05 (0.85–1.29)	0.312	14.2	1.03 (0.53–2.01)	0.592	0
All ethnicity	5	1.22 (1.05–1.42)	0.035	61.2	1.46 (0.92–2.32)	0.558	0.0	1.26 (1.05–1.50)	0.016	67.1	1.26 (1.07–1.50)	0.011	69.2	1.26 (0.80–1.99)	0.790	0.0

*CI, confidence interval, OR, odds ratio, I^2^ = the variation in OR attributable to heterogeneity.*

**FIGURE 4:**
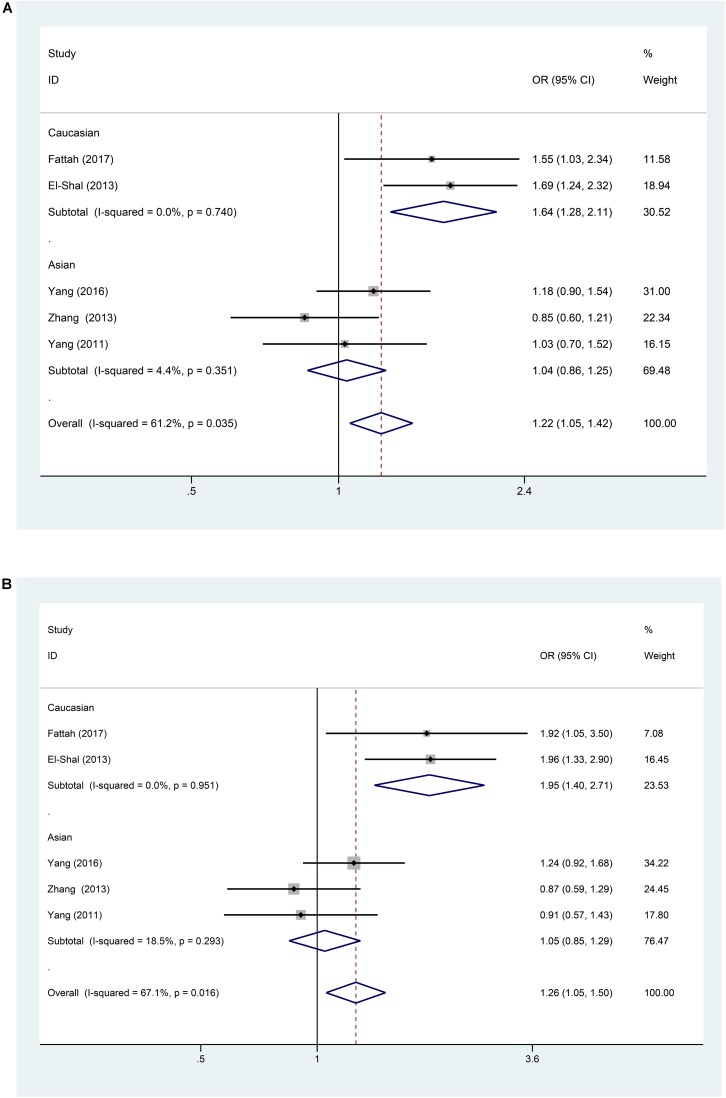
**(A)** Forest plot for association between miR-499a rs3746444 polymorphisms and risk of rheumatoid arthritis under allele genetic model (C vs. T) after stratification analysis by ethnicity. **(B)** Forest plot for association between miR–499a rs3746444 polymorphisms and risk of rheumatoid arthritis under heterozygote genetic model (TC vs. TT) after stratification analysis by ethnicity.

**FIGURE 5:**
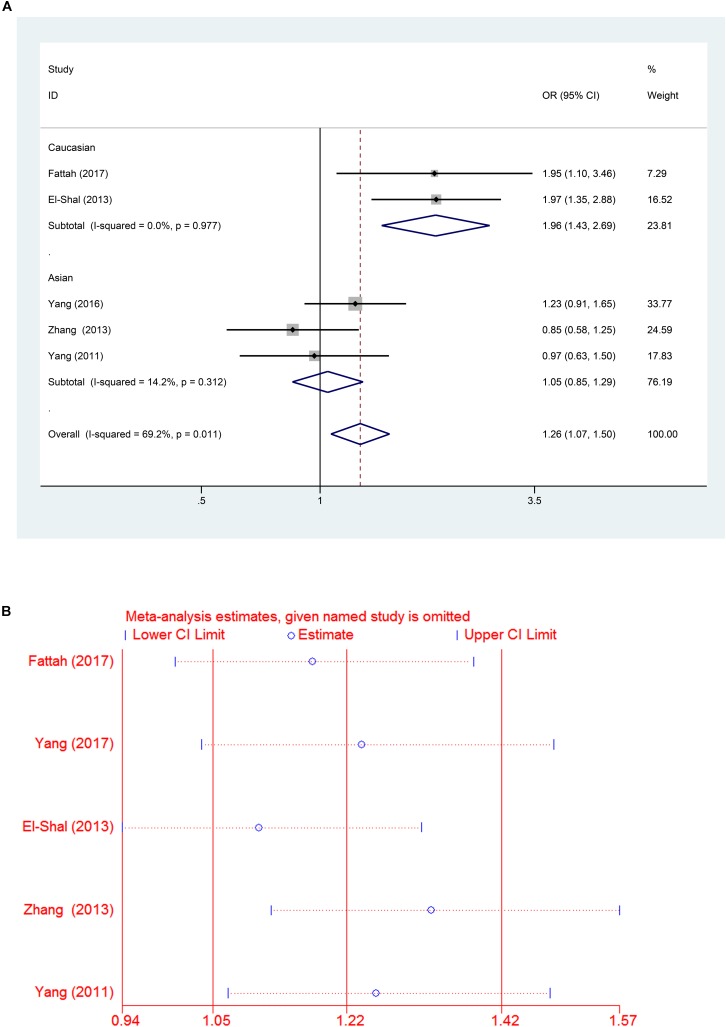
**(A)** Forest plot for association between miR-499a rs3746444 polymorphisms and risk of rheumatoid arthritis under dominant genetic model (TC/CC vs. TT) after stratification analysis by ethnicity. **(B)** The sensitivity analysis for studies of the association between miR–499a rs3746444 polymorphisms and risk of rheumatoid arthritis under allele genetic model (C vs. G).

A significant association between the miR-499 rs3746444 T > C polymorphism and RA risk was observed in all populations, as shown in Table 2 (C vs. T: OR = 1.22, 95% CI = 1.05–1.42, P_h_ = 0.035; TC vs. TT: OR = 1.26, 95% CI = 1.05–1.50, P_h_ = 0.016; TC/CC vs. TT: OR = 1.26, 95% CI = 1.07–1.50, P_h_ = 0.011). However, no significant associations were found in CC vs. TT: OR = 1.46, 95% CI = 0.92–2.32, *P* = 0.558 and CC vs. TC/TT: OR = 1.26, 95% CI = 0.8–1.99, *P* = 0.79 (all *P* > 0.05, Table 2). Subgroup analysis based on ethnicity showed no significant association between the miR-146a C/T polymorphism and susceptibility to RA in the Asian population (*P* > 0.05) (Table 2). However, in the Caucasian population, the C allele in the miR-499 T > C polymorphism was a contributor to RA susceptibility in some genetic models (C vs. T: OR = 1.64, 95% CI = 1.28–2.11, P_h_ = 0.74; TC vs. TT: OR = 1.95, 95% CI = 1.40–2.71, P_h_ = 0.951; TC/CC vs. TT: OR = 1.96, 95% CI = 1.43–2.69, P_h_ = 0.977) (all *P* < 0.001, Table 2 and Figures 4A,B, 5A).

### Sensitivity Analysis and Publication Bias

A leave-one-out analysis was performed to investigate the influence of each individual study on the pooled ORs (Figures 3B, 5B). The results indicated no significant alteration in the pooled ORs after exclusion of any study on miR-146a rs2910164 G > C and miR-499 rs3746444 T > C. Moreover, because of the limited number of studies included (<10), publication bias was not evaluated.

## Discussion

Genetic and epigenetic factors play pivotal roles in cancer and autoimmune diseases. MiRNAs are important for the regulation of the cartilage-invading phenotype of RA synovial fibroblasts, leukocyte activation, and cytokine production, and their roles contribute to our understanding of the mechanisms underlying RA and provide novel therapeutic targets for the treatment of RA (Baxter et al., 2012).

MiR-146a, one of the most extensively investigated miRNAs, exerts significant effects on cancer and autoimmune diseases. MiR-146a is widely expressed and involved in the differentiation and activation of the innate and adaptive immune systems (O’Connell et al., 2010). It suppresses NF-κB signaling by downregulating the NF-κB signaling transducers interleukin-1 (IL-1) receptor associated kinase 1 and tumor necrosis factor receptor-associated factor 6. Meanwhile, miR-146a directly activates signal transducer and activator of transcription 1 to promote the activity of Treg cells in RA, which mediate the main mechanisms underlying RA (Zhou Q. et al., 2015). Polymorphisms of miR-146a are associated with various cancers and autoimmune diseases (Wang et al., 2012; Xu et al., 2012).

A single nucleotide polymorphism consisting of the G/C nucleotide substitution in miR-146a rs2910164 could result in the replacement of a G:U pair by a C:U mismatch (Hassine et al., 2017). However, the association between mir146a polymorphisms and RA remains controversial. Because of the increasing number of studies, we set strict inclusion and exclusion criteria to avoid potential bias from inadequate studies. Data from Singh et al. (2014). were not included in this meta-analysis because the patients were diagnosed as juvenile idiopathic arthritis (JIA), which includes other types of arthritis in young patients. Data from El-Shal et al. (2013) were also excluded from the pooled analysis for mir146a because of deviation from HWE in the control group. The results of the updated meta-analysis suggest that mir146a polymorphisms are not associated with RA risk, which is consistent with previous studies (Li et al., 2014; Lee and Bae, 2015).

The mechanism underlying the association between RA and miR-499 was investigated previously. One possible explanation is that mir-499 modulates the expression of regulatory factor X4, thus regulating the human leukocyte antigen DRB1, which is closely associated with RA (van Gaalen et al., 2004; Yang et al., 2011). Moreover, miR-499 targets IL-17 receptor B, IL-6, and several cytokines, which are important factors involved in RA pathogenesis (Ying et al., 2011; El-Shal et al., 2013). Although previous studies demonstrated the association between miR-499 polymorphisms and RA, the limited study number and high heterogeneity decreased the robustness of the conclusions. In the present study, two studies from the miR-499 polymorphism group were excluded from the meta-analysis because the genotype distribution of the controls in all studies was inconsistent with HWE (Hashemi et al., 2013; Toraih et al., 2016). Fattah et al. (2017) found that miR-499 rs3746444 T > C is associated with the susceptibility to RA, and the heterozygote is characterized by higher levels of C-reactive protein, anti-cyclic citrullinated peptide, antibody and disease activity score 28 than those of the homozygote (Fattah et al., 2017). Hashemi et al. (2013) also reported that the C allele of the mir-499 rs3746444 polymorphism is a risk factor for RA. Although the overall results of the pooled analysis indicated that the miR-499 T > C polymorphism was associated with RA risk, the high heterogeneity suggested an insufficient robustness of the conclusion. In the subgroup analysis according to ethnicity, a strong correlation between the miR-499 T > C polymorphism and RA was observed in the Caucasian group, which suggested that the C allele in the miR-499 T > C polymorphism was an obvious contributor to RA susceptibility. However, no relationship was observed in the Asian group. The results were in consistency with previous meta-analysis (Fu et al., 2016), however, Fu et al. (2016) drew a conclusion about miR-499 polymorphism group based on three studies including two studies (El-Shal et al., 2013; Hashemi et al., 2013) from the Caucasian group and one (Yang et al., 2011) from the Asian group. As increasing number of case-control studies published, this updated meta-analysis retrieved five studies including two (El-Shal et al., 2013; Fattah et al., 2017) from the Caucasian group and three from Asian group (Yang et al., 2011, 2016; Zhang et al., 2013). Due to the inconsistency with HWE, two studies (Hashemi et al., 2013; Toraih et al., 2016) were excluded from the miR-499 polymorphism group, which increased the robustness of this meta-analysis.

The present study had several limitations. Firstly, the variation in sample size and in genotyping methods was a limitation of the study. Secondly, all studies included in our meta-analyses were in accordance with HWE, and because of the limited number of included studies, no publication bias could be tested. Because of the limited quantity and quality of the included studies, the above conclusion needs to be further verified in additional high quality studies.

## Conclusion

In conclusion, the present meta-analysis showed no significant association between miR-146a and RA risk, which is consistent with previous studies. However, the present study demonstrated the contribution of the C allele in the miR-499 T > C polymorphism to RA susceptibility in the Caucasian population, and this association was not present in the Asian population.

## Author Contributions

XZ conceived and designed the study and contributed to statistical analyses. MZ, BJ, and MX contributed to eligible study collection and data extraction. XZ and BJ prepared the tables and figures. XZ, MZ, and MX wrote and revised the manuscript.

## Conflict of Interest Statement

The authors declare that the research was conducted in the absence of any commercial or financial relationships that could be construed as a potential conflict of interest.
